# Discovery of C—C bond-forming and bond-breaking radical enzymes: enabling transformations for metabolic engineering

**DOI:** 10.1016/j.copbio.2020.02.003

**Published:** 2020-10

**Authors:** Beverly Fu, Emily P Balskus

**Affiliations:** Department of Chemistry and Chemical Biology, Harvard University, 12 Oxford St., Cambridge, MA 02138, United States

## Abstract

Radical enzymes catalyze some of the most chemically challenging C—C bond-forming and bond-breaking reactions. Advances in DNA sequencing have accelerated the discovery of radical enzymes from microbes, including radical *S*-adenosylmethionine (rSAM) enzymes, glycyl radical enzymes (GREs), and diiron enzymes. These enzymes catalyze various reactions that yield products of industrial relevance (e.g. aromatics, hydrocarbons, and natural product derivatives), making their incorporation into engineered metabolic pathways enticing. Elucidating the mechanisms of radical enzymes that cleave and construct C—C bonds will enable further enzyme discovery and engineering efforts.

## Highlights

• Radical enzymes catalyze difficult C—C bond-forming and bond-breaking transformations.
• Radical enzymes catalyzing unprecedented reactions continue to be discovered.
• The products of radical enzymes are often of high value.
• Understanding mechanisms of radical enzymes will aid metabolic engineering efforts.

## Graphical abstract

**Figure:**
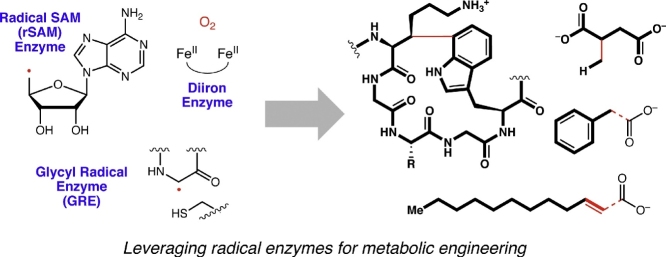

**Current Opinion in Biotechnology** 2020, **65**:94–101

This review comes from a themed issue on **Chemical Biotechnology**

Edited by **Christoph Wittmann** and **Sang Yup Lee**

For a complete overview see the Issue and the Editorial

Available online 1st April 2020

**https://doi.org/10.1016/j.copbio.2020.02.003**

0958-1669/© 2020 The Authors. Published by Elsevier Ltd. This is an open access article under the CC BY license (http://creativecommons.org/licenses/by/4.0/).

## Introduction

The declining cost of DNA sequencing has resulted in an abundance of genomes and metagenomes. Characterizing the functions of the proteins encoded within this vast reservoir of genomic information is now an enormous bottleneck. For example, in the healthy human gut microbiota, roughly 50% of gene sequences cannot be annotated [1], and enzymes with putative functional assignments often only have broad annotations that cannot accurately predict activity. Many uncharacterized microbial genes likely encode previously unappreciated enzymes; discovering these enzymes will improve current annotation methodologies and also reveal activities that may be leveraged for metabolic engineering.

**Figure 1:**
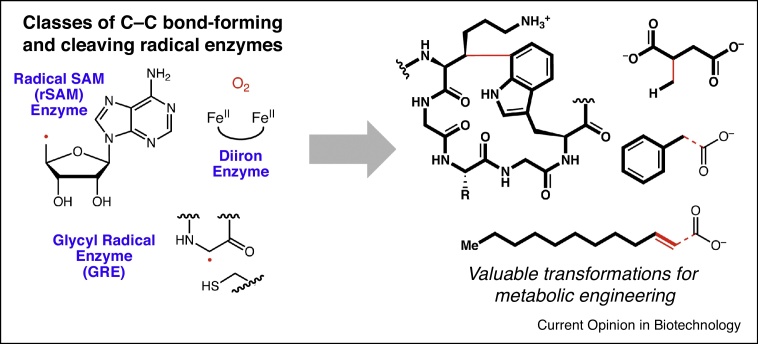
Radical enzymes, including radical *S*-adenosylmethionine (rSAM) enzymes, glycyl radical enzymes (GREs), and diiron enzymes, perform various challenging C—C bond-forming and bond-breaking reactions that have the potential to be incorporated into engineered metabolic pathways. Examples of such products are shown on the right. The C—C bonds formed and/or broken by radical enzyme action are highlighted by the red solid and dashed lines, respectively. The production of these metabolites requires critical radical enzyme cofactors depicted on the left. Understanding the mechanisms of these enzymes will improve our ability to leverage them to their utmost capacity.

Radical enzymes are of particular interest from this perspective. Radical enzymes utilize protein-based and cofactor-based radical intermediates to catalyze challenging chemical transformations that cannot always be accomplished via standard two-electron mechanisms [2]. In particular, C—C bond formation at unactivated carbon centers and cleavage of unreactive C—C bonds is difficult to achieve by non-radical pathways. Examples of radical enzymes that enable these types of transformations include radical *S*-adenosylmethionine (rSAM) enzymes, glycyl radical enzymes (GREs), and diiron enzymes (Figure 1). These classes of enzymes have characteristic sequence motifs, enabling their identification.

There are many untapped, enabling transformations to be discovered among these radical enzyme families, and their incorporation into engineered metabolic pathways may allow the production of high value chemicals, such as biofuels, aromatics, and natural product derivatives, from cheap, renewable feedstocks. This review highlights the discovery and proposed mechanisms of recently identified C—C bond-forming and bond-breaking radical enzymes that may enable metabolic engineering.

## C—C bond formation: radical SAM enzymes involved in RiPP biosynthesis

Ribosomally synthesized and post-translationally modified peptides (RiPPs) are a diverse class of natural products that have a variety of bioactivities including disrupting protein-protein interactions [3]. During RiPP biosynthesis, a genetically encoded linear peptide precursor is extensively post-translationally modified to add structural complexity and imbue functions of target recognition and metabolic and chemical stability. This biosynthetic logic makes RiPPs amenable to combinatorial screening for new bioactivities. Previous efforts to engineer RiPP pathways include activity-based screening of lanthipeptide libraries generated using a promiscuous lanthipeptide synthetase, ProcM, for peptides that could disrupt a protein–protein interaction necessary for HIV budding [4].

Continued work in developing RiPP-based therapeutic leads relies on understanding how tailoring enzymes from these pathways function. Radical *S*-adenosylmethionine (rSAM) enzymes are among the most prolific RiPP tailoring enzymes and are responsible for many characteristic structural modifications including thioether bridge and carbon–carbon bond formation, methylation, epimerization, and complex rearrangements [5].

The rSAM enzyme superfamily is one of the largest, with 400,000 members identified to date [6,7]. These enzymes use a [4Fe–4S]^+^ cluster to reductively cleave SAM, generating a transient 5′-deoxyadenosine radical (5′-dA•) species that can abstract a hydrogen atom (H-atom) from the substrate, initiating radical chemistry that can lead to C—C bond formation. Many members of this superfamily contain a SPASM/Twitch domain, a C-terminal extension that can bind up to two auxiliary [Fe—S] clusters [8]. The roles of these clusters are not yet clear, although they have proposed roles in electron transfer.

**Figure 2:**
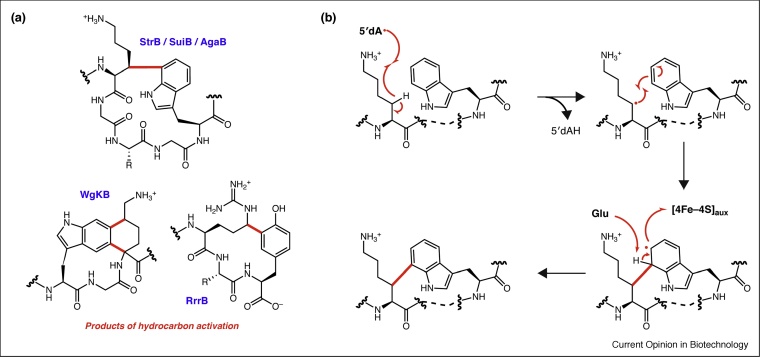
C—C bond-forming radical *S*-adenosylmethionine (rSAM) enzymes are involved in tailoring a class of natural products known as ribosomally synthesized and post-translationally modified peptides (RiPPs). **(a)** Recently discovered rSAM enzymes StrB, SuiB, and AgaB catalyze C—C bond formation between Lys and Trp. Similarly, WgkB catalyzes the formation of an unprecedented two C—C bonds between Trp and Lys, while RrrB links Lys and Tyr. **(b)** The current mechanistic proposal for StrB involves H-atom abstraction from the β-carbon of Lys. Electrophilic and deprotonation by a nearby Glu yields a radical anion which quickly re-aromatizes.

During the past five years, multiple novel rSAM enzymes involved in RiPP biosynthesis have been discovered (Figure 2a). For instance, streptides, a new class of RiPPs with an unusual Lys-Trp crosslink (β-C of Lys and C7 of Trp), were identified in 2015 [9]. The enzyme responsible for installing this key feature is the rSAM enzyme StrB. This SPASM-domain containing rSAM enzyme requires an auxiliary [4Fe—4S] cluster for catalysis. Initial mechanistic studies using perdeuterated Lys showed H-atom abstraction occurs from the Lys side chain [9]. StrB homologs from two human pathogens, *Streptococcus agalactiae* (AgaB) and *Streptococcus suis* (SuiB), were also shown to catalyze the same reaction [10] with similar kinetic parameters (StrB *k*_cat_ = 0.0023 s^−1^, *K*_M_ = 160 μM [9]; AgaB *k*_cat_ = 0.002 s^−1^, *K*_M_ = 50 μM; and SuiB *k*_cat_ = 0.003 s^−1^ [11^•^]). Through testing of substrate analogs, it was shown that the amino group of Lys is necessary for catalysis, potentially playing a role in orienting substrate through a salt bridge or hydrogen bond or alternatively by acting as a Brønsted base.

Follow-up studies focusing on AgaB and SuiB explored whether a radical addition or electrophilic aromatic substitution mechanism is employed [11^•^]. Substrate analogs varying the Lys side chain showed that length rather than p*K*_a_ impacts turnover, thus providing support for this residue having an anchoring role. Notably, a crystal structure of SuiB implicates another nearby Glu as a candidate general base. Mutation of the Glu to Ala resulted in an eightfold lower *k*_cat_ with minimal change in *K*_M_. Altogether, a radical electrophilic aromatic substitution mechanism was proposed involving the formation of a radical anion that rearomatizes via one electron donation to the auxiliary [4Fe—4S] cluster, as summarized in Figure 2b.

Bioinformatic searches revealed 600 unexplored rSAM enzyme-encoding RiPP gene clusters under the control of regulatory systems similar to that of the streptide-encoding gene cluster. The first of these rSAM enzymes to be explored was WgkB, which links the side-chains of Trp and Lys by forming two C—C bonds, resulting in a substituted tetrahydro[5,6]benzindole moiety [12^•^]. More recently, RrrB was shown to catalyze C—C bond formation between the *δ*-carbon of an Arg side chain to the ortho-position of a tyrosine-phenol in the assembly of ryptides [7]. Unlike previously characterized C—C bond-forming rSAM enzymes, a carbon-center adjacent to a heteroatom undergoes coupling in this reaction. To explore RrrB promiscuity, different peptide substrates were tested. While conservative changes, such as Arg to Lys or Tyr to Phe, were not accepted, truncated substrates were processed. The biological roles of these new RiPPs have yet to be elucidated. However, as they are all under quorum-sensing regulation and are structurally unique, they may have interesting bioactivities.

In addition to these RiPP-associated rSAMs, cobalamin B_12_-dependent rSAM enzymes install methyl groups onto unactivated carbon centers of Trp and Val in other RiPP pathways [5], and have also recently been shown to catalyze C—O bond formation (TqqB Thr-Gln [13]) and C—S bond formation (NxxcB Cys-Asn [14], CteB Cys-Thr [15]). A deeper understanding of how these rSAM enzymes catalyze reactions will aid in natural product engineering efforts.

## C—C bond formation: hydrocarbon activating glycyl radical enzymes

Aromatic hydrocarbons, such as toluene, are present in groundwater as a consequence of natural processes and human pollution. These molecules are resistant to oxidation and de-aromatization, making bioremediation difficult. However, various microbes have evolved anaerobic pathways for hydrocarbon degradation that employ glycyl radical enzymes (GREs). This family of O_2_-sensitive enzymes uses a conserved glycine-centered radical to catalyze difficult chemical transformations [16]. The stable, protein-based glycyl radical is post-translationally installed by a dedicated partner activating enzyme (GRE-AE) belonging to the rSAM enzyme superfamily. The transient 5′-dA• species generated by the GRE-AE abstracts an H-atom from a conserved GRE backbone glycine. The glycyl radical is hypothesized to generate a thiyl radical on a conserved cysteine residue, and this proposed intermediate is thought to react with substrate. Further reaction or rearrangement of a substrate-based radical, followed by consecutive H-atom transfers from the cysteine and glycine, provide product and regenerate the glycyl radical.

**Figure 3:**
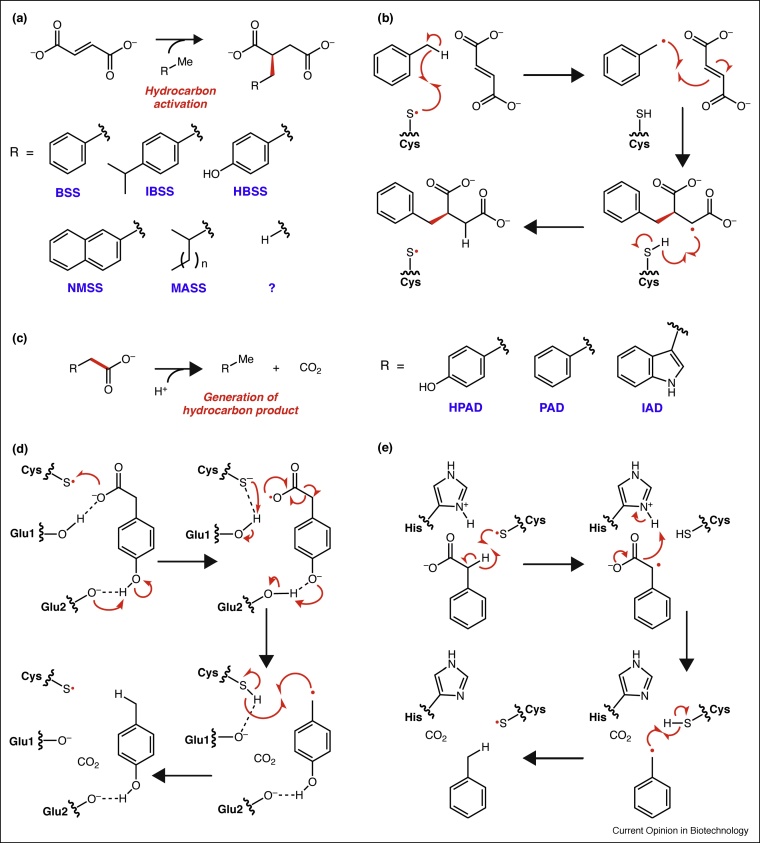
C—C bond-forming glycyl radical enzymes (GREs) are involved in the bioremediation of aromatic hydrocarbons while C—C bond-breaking GREs generate small aromatic hydrocarbons that are potential fuels and fragrances. **(a)** Although BSS, which catalyzes the addition of toluene to fumarate, is the most well characterized of these C—C bond-forming GREs, there is evidence that related enzymes metabolize other aromatic hydrocarbons (see text for abbreviations). In addition, it has been postulated that a similar GRE may be involved in anaerobic methane oxidation. **(b)** The current mechanistic proposal for BSS involves H-atom abstraction from toluene, generating a benzylic radical. Electrophilic addition to fumarate generates a radical at the C3 position. Another H-atom abstraction generates product and regenerates the protein-based radical. **(c)** HPAD, PAD, and IAD catalyze decarboxylation reactions to produce *p*-cresol, toluene, and skatole, respectively. **(d)** HPAD is proposed to perform a Kolbe-type decarboxylation reaction. Carboxylate oxidation coupled to deprotonation of the phenolic oxygen generates the substrate radical. Decarboxylation then occurs, coupled to re-protonation of the phenolate. Another H-atom abstraction generates product and regenerates the protein-based radical. **(e)** In contrast, PAD is proposed to generate the substrate-based radical via H-atom abstraction of the methylene. Decarboxylation and protonation by a nearby residue then follows, providing a benzylic radical.

A subset of GREs, collectively known as the x-succinate synthases, catalyze C—C bond formation between a variety of unactivated hydrocarbons and fumarate, enabling further metabolism (Figure 3a). Many of these enzymes are well documented (benzylsuccinate synthase (BSS), 4-isopropylbenzylsuccinate synthase (IBSS), hydroxybenzylsuccinate synthase (HBSS), naphthyl-2-methylsuccinate synthase (NMSS), 1-methylalkylsuccinate synthase (MASS) [16]). Because of the oxygen sensitivity of these enzymes, *in vitro* kinetic parameters are difficult to assess, but BSS is estimated to have a *K*_M_ of <100 μM and *V*_max_ of 7.4 nmol min^−1^ mg^−1^ [17]. Recently solved crystal structures of BSS provide evidence for the proposed mechanism (Figure 3b) and modulation of enzyme activity through subunit conformational dynamics [18,19]. In addition, a putative GRE is proposed to be involved in anaerobic oxidation of methane by catalyzing the addition of methane to fumarate [20,21]. Continued efforts to discover and study related enzymes may inform future bioremediation efforts. Although aerobic microbial hydrocarbon degradation pathways exist, their dependence on O_2_ makes them unsuitable for use in soils that have anaerobic zones [22,23].

## C—C bond cleavage: decarboxylating glycyl radical enzymes

GREs also catalyze C—C bond cleavage reactions, including decarboxylation. The three GRE decarboxylases characterized to date, hydroxyphenylacetate decarboxylase (HPAD) [24], phenylacetate decarboxylase (PAD) [25], and indoleacetate decarboxylase (IAD) [26], catalyze the conversion of *p*-hydroxyphenylacetate, phenylacetate, and indoleacetate into *p*-cresol, toluene, and skatole, respectively (Figure 3c). Each of these products has commercial uses. *p*-Cresol is an important raw material for producing chemicals such as the antioxidant butylated hydroxytoluene [27]. 60% of the American, European, and Japanese supply of *p*-cresol is from synthetic sources, while 40% is from natural sources such as coal tar [27]. Toluene is an important petrochemical that has a global market of 29 million tons/year, and it is used to synthesize other aromatic feedstocks. Finally, while skatole is most commonly associated with the livestock diseases fog fever and boar taint, it is also an additive in perfumes and other aromatics. It is accessed synthetically via Fischer indole synthesis [28].

These C—C bond-cleaving GREs were each discovered using different methods [29]. HPAD was found via activity-guided fractionization of *Clostridioides difficile*, a known *p*-cresol producer. PAD was discovered from two anoxic, toluene-producing microbial communities: municipal sewage sludge and lake sediments from Berkeley, CA. Considering functional similarities to HPAD, PAD was postulated to be a GRE. Through activity-guided protein fractionation and metagenomic and metaproteomic data analysis of active fractions, a putative GRE decarboxylase was identified. *In vitro* experiments showed that this enzyme was indeed PAD [25]. IAD was recently discovered by comparing GREs encoded in the genomes of skatole-producing and non-producing bacterial strains [26].

Although each GRE catalyzes a conceptually similar decarboxylation reaction, it is presently unclear whether they employ related mechanisms (Figure 3d versus e). HPAD is proposed to use a Kolbe-type decarboxylation strategy where the thiyl radical oxidizes the carboxylate group by one electron, generating a carboxyl radical. This step is proposed to be coupled to substrate deprotonation at the phenolic position, resulting in a de facto H-atom transfer. Re-protonation of the phenolate is proposed to drive decarboxylation (Figure 3d). The HPAD mechanism is supported by the orientation of *p*-hydroxyphenylacetate in the active site of a crystal structure [30] as well as computational studies [31]. PAD is believed to employ a different decarboxylation strategy due to the lack of a *para*-hydroxyl group. Instead of oxidizing the carboxylate, the thiyl radical is postulated to abstract an H-atom from the α-methylene. Heterolytic decarboxylation of the resulting benzylic radical, protonation by a nearby His (identified only through homology modelling), and H-atom abstraction from the Cys yields toluene [32^••^] (Figure 3e). This proposal was derived from computational calculations as well as studies using a substrate analog. As for IAD, homology modeling identified conserved residues consistent with a Kolbe-type decarboxylation mechanism [26], but further characterization is necessary to test this proposal. The fact that two distinct mechanisms have been proposed raises questions about how these GRE decarboxylases evolved and highlights the need for additional studies.

Applying these enzymes in metabolic engineering would provide access to small aromatic products from amino acid precursors. However, a major obstacle to be addressed is low enzyme activity; for example, PAD’s activity is very low (*k*_cat_ = 7.5 × 10^−7^ s^−1^, *K*_M_ = 2.54 mM [32^••^]), potentially due to poor enzyme solubility. The other two decarboxylases have much higher turnover numbers comparable to those of other GREs (HPAD’s *k*_cat_ = 110 s^−1^, *K*_M_ = 0.65–2.8 mM [24,33]; IAD’s *k*_cat_ = 2 s^−1^, *K*_M_ = 0.37 mM [26]). A better understanding of mechanism could enable engineering efforts to enhance solubility, improve catalytic rates, and expand substrate scope. Other GREs have been used in engineered metabolic pathways, most notably glycerol dehydratase (GD), which catalyzes the dehydration of glycerol, 1,2-propanediol, and 1,2-ethanediol to give 3-hydroxypropionaldehyde (3HPA), propionaldehyde, and acetaldehyde, respectively [34]. 3HPA can be converted into a variety of lucrative compounds including 3-hydroxypropionic acid (precursor for acrylic acid, bioplastics) [34] and 1,3-propanediol (biodegradable polymers) [35]. Importantly, GREs could replace coenzyme B_12_-dependent GDs that are employed in related processes, reducing cost by eliminating the need for added B_12_ cofactor [35, 36, 37]. Analogous incorporation of C—C bond-forming and C—C bond-cleaving GREs in engineered metabolic pathways would expand the types of compounds attainable using this approach.

## C—C bond cleavage: hydrocarbon-forming diiron enzymes

**Figure 4:**
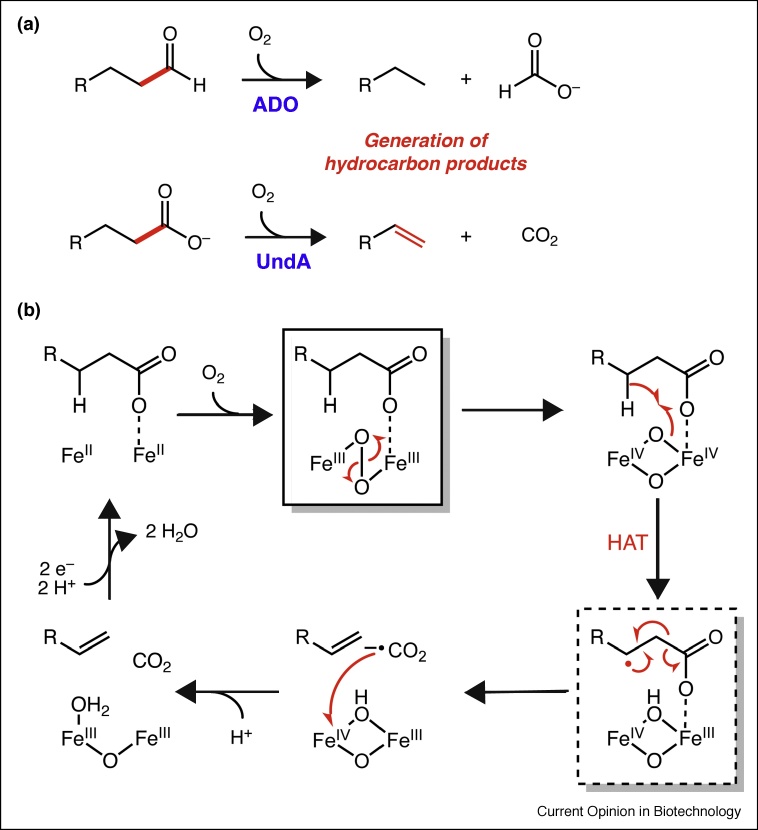
C—C bond-breaking diiron enzymes produce medium chain aliphatic hydrocarbons. **(a)** ADO and UndA process aldehydes or carboxylic acids, generating formate or carbon dioxide as byproducts. **(b)** UndA was recently shown to be a diiron enzyme. The current mechanistic proposal involves O_2_ activation, leading to a characterized μ-peroxo-Fe_2_(III/III) intermediate (boxed). This intermediate is then postulated to convert to an H-atom abstracting Fe_2_(IV/IV) complex. Substrate oxidation then leads to the Fe_2_(III/IV) intermediate also observed spectroscopically (dashed box). Multiple plausible mechanisms for the subsequent decarboxylation reaction have been proposed, one of which is depicted here.

Diiron enzymes catalyze C—C cleavage reactions through oxidative and oxygenative mechanisms. For example, aldehyde-deformylating oxygenase (ADO) uses molecular oxygen to catalyze C1—C2 bond cleavage of free saturated or monounsaturated C_n_ (*n* = 16, 18, 20) fatty aldehydes, generating the corresponding C_n−1_ hydrocarbon that can be used as petrodiesel [38]. In the same vein, another recently verified diiron enzyme (UndA) catalyzes the decarboxylation of free C_x_ (*x* = 10, 12, 14) fatty acids to C_x−1_ terminal olefins that can be directly employed as liquid fuel or industrial feedstocks [39] (Figure 4a). Both of these hydrocarbon-forming enzymes have been incorporated in engineered metabolic pathways in cyanobacteria [40,41]. In general, diiron enzymes use a cofactor containing two-coupled Fe^II^ ions ligated by protein residues (Asp, Glu, His) [42]. This reduced form reacts with molecular oxygen, resulting in a variety of potential oxidized cofactor and reduced oxygen intermediates that can oxidize unactivated substrates.

UndA was first identified by screening a fosmid library from various *Pseudomonas* strains known to produce 1-undecene [39]. *In vitro* biochemical characterization showed it converts medium-chain fatty acids (C10, C12, C14) into the corresponding terminal olefins. Under conditions of multiple turnover using ascorbate as an external reductant, the rate for UndA *in vitro* (0.0011 s^−1^) was much lower than the initial rates under single turnover conditions (0.06 s^−1^). Nevertheless, simple overexpression of UndA homologs in *Escherichia coli* led to titers 25-fold higher than in *Pseudomonas* [39]. The initial crystal structure of *Pseudomonas fluorescens* Pf-5 UndA had only a single Fe atom bound in close proximity to the β-H-atom of a substrate analog, leading to the proposal that it was a mononuclear non-heme Fe enzyme [39]. More recently, UndA from *P. syringae pv. tomato* was crystalized and found to have conserved ligands consistent with binding two Fe atoms, although no Fe was bound in the crystal structure [43]. However, Mössbauer spectroscopy of the reconstituted enzyme showed the presence of a magnetically-coupled dinuclear iron center.

Additional experiments with the *P. fluorescens* UndA corroborated these findings [44^•^]. In this case, the crystal structure was solved with better Fe occupancy, but not all of the ligands could be fully modeled. Mutation of each ligand to Ala abolished activity. Moreover, an on-pathway reaction intermediate that forms upon O_2_ activation was detected and determined to be a μ-peroxo-Fe_2_(III/III) complex using a combination of stopped-flow UV–vis and freeze-quench Mössbauer spectroscopy. An Fe_2_(III/IV) complex also accumulated in this experiment although it is unclear if this species is on pathway. One current mechanistic proposal is reported in Figure 4b. Exploration of alternative hosts and feedstocks along with continued dissection of mechanism of UndA and related enzymes could increase catalytic efficiencies, which is currently a bottleneck in improving product titers [45].

## Conclusions and future prospects

There is great utility in continuing to discover and study radical enzymes, as they catalyze an abundance of difficult chemical transformations not readily achieved by traditional synthetic chemistry. Many radical enzymes already produce compounds of great value (e.g. hydrocarbons produced by diiron enzymes), and future engineering efforts could further expand the scope of products accessed as well as improve enzyme activity. Realizing the potential of radical enzymes in engineered pathways will require continued progress in elucidating the mechanisms of characterized enzymes and expanded efforts to discover new family members.

## Conflict of interest statement

Nothing declared.

## References and recommended reading

Papers of particular interest, published within the period of review, have been highlighted as:

• of special interest
•• of outstanding interest
